# A Protective Role of Paeoniflorin in Fluctuant Hyperglycemia-Induced Vascular Endothelial Injuries through Antioxidative and Anti-Inflammatory Effects and Reduction of PKC*β*1

**DOI:** 10.1155/2019/5647219

**Published:** 2019-04-10

**Authors:** Jing-Shang Wang, Ye Huang, Shuping Zhang, Hui-Jun Yin, Lei Zhang, Yan-Hong Zhang, Ye-Wen Song, Dan-Dan Li

**Affiliations:** ^1^Department of Traditional Chinese Medicine, Beijing Obstetrics and Gynecology Hospital, Capital Medical University, Beijing 100026, China; ^2^Department of Andrology, Dongzhimen Hospital, Beijing University of Chinese Medicine, Beijing 100007, China; ^3^Emergency Department, Xiyuan Hospital, China Academy of Chinese Medical Sciences, Beijing 100091, China; ^4^Institute for Medical Engineering and Science, Massachusetts Institute of Technology, Cambridge, MA 02139, USA; ^5^Department of Cardiovascular Disease, Xiyuan Hospital, China Academy of Chinese Medical Sciences, Beijing 100091, China

## Abstract

Hyperglycemia fluctuation is associated with diabetes mellitus (DM) complications when compared to persistent hyperglycemia. Previous studies have shown that paeoniflorin (PF), through its antiapoptosis, anti-inflammation, and antithrombotic properties, effectively protects against cardiovascular and cerebrovascular disease. However, the mechanism underlying the protection from PF against vascular injuries induced by hyperglycemia fluctuations remains poorly understood. Herein, we investigated the potential protective role of PF on human umbilical vein endothelial cells (HUVECs) subjected to intermittent glucose levels *in vitro* and in DM rats with fluctuating hyperglycemia *in vivo*. A remarkable increased apoptosis associated with elevated inflammation, increased oxidative stress, and high protein level of PKC*β*1 was induced in HUVECs by intermittently changing glucose for 8 days, and PF recovered those detrimental changes. LY333531, a potent PKC*β*1 inhibitor, and metformin manifested similar effects. Additionally, in DM rats with fluctuating hyperglycemia, PF protected against vascular damage as what has been observed *in vitro*. Taken together, PF attenuates the vascular injury induced by fluctuant hyperglycemia through oxidative stress inhibition, inflammatory reaction reduction, and PKC*β*1 protein level repression, suggesting its perspective clinical usage.

## 1. Introduction

Diabetes mellitus (DM) is a metabolic disorder, the fifth leading cause of death worldwide, accounting for 5.2% of all deaths [1]. Recent studies have shown that both increased glucose levels and glucose level fluctuations are closely related to the occurrence and development of DM complications [2]. Compared to chronic persistent hyperglycemia, fluctuant hyperglycemia can easily trigger endothelial cell dysfunction and vascular complications [3], but the exact underlying mechanisms are still unknown. Accumulated evidence indicates that increased vascular oxidative stress and inflammation are associated with vascular dysfunction in DM [4], whereas fluctuant hyperglycemia is more effective than constant hyperglycemia in inducing the apoptosis of human umbilical vein endothelial cells (HUVECs) [5] and the oxidative stress [6] and inflammation [7] in human coronary artery endothelial cells [8]. The high intracellular glucose levels in hyperglycemia cause overactivation of various biochemical pathways [9]. Of them, the protein kinase C (PKC) activation pathway, one of the biochemical mechanisms for the progression of diabetes-associated complications [10] is involved in vascular permeability, extracellular matrix synthesis, cell growth, cytokine activation, angiogenesis, ROS production, and vascular smooth muscle contractility [9, 11]. PKC*β*, an isozyme of PKC, appears to play a critical role in the pathogenesis of microvascular complications [12, 13]. The microarray results from our previous study identified that PKC*β*1 was one of the genes expressed in peripheral leukocytes with higher expression in coronary heart disease (CHD) patients compared to the healthy control [14]. Since CHD is the leading cause of morbidity and mortality in patients with DM [15], we assume that high levels of PKC*β*1 might be involved in glucose fluctuation.

**Figure 1:**
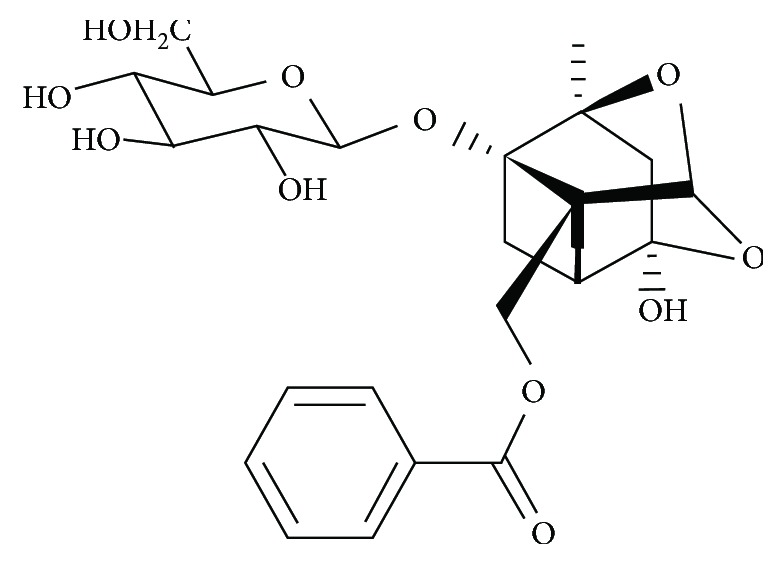
Chemical structure of PF.

Paeoniflorin (PF, Figure 1) is an important component of *Paeonia lactiflora* Pall that shows various protective effects on the cardio-cerebral vascular system, mediated by antiapoptosis [16], anti-inflammation [17], and antioxidantation [18]. Furthermore, PF is reported to play an essential protective role in both DM-associated macrovascular complications, such as myocardial infarction via the transient receptor potential vanilloid 1/calcitonin gene-related peptide pathway [19], and DM-associated microvascular complications, such as diabetic nephropathy with renal protective effects by prevention of TLR2/4-mediated inflammation [20] or inhibition of the JAK2/STAT3 signaling pathway [21]. However, the effects and mechanisms of PF on glucose fluctuation-induced damages remain to be elucidated. Metformin is a potent antihyperglycemic agent which is widely used in the management of type 2 diabetes by suppressing gluconeogenesis and improving glucose uptake and insulin sensitivity [22]. Moreover, previous studies have shown that metformin improves vascular functions and dramatically reduces the incidence of vascular complications through improving glycemic control, insulin resistance, lipid profile, fibrinolitic activity, oxidative stress, and endothelial function [23]. However, some adverse effects of metformin such as digestive tract symptoms including diarrhea, flatulence, and abdominal discomfort limited its application in clinics [22]. Thus, additional effects should be made in developing new drugs for treatment of DM. Therefore, metformin was used as a positive control to assess the effect of PF on vascular endothelial injury, inflammation, and oxidative stress under intermittent hyperglycemia.

This study is aimed at unveiling the potential protective role of PF in intermittent glucose-induced vascular endothelial injuries using HUVECs and a rat model of hyperglycemia fluctuation under different glycemic conditions. Inflammatory markers, oxidative stress indexes, and PKC*β*1 protein levels were measured by ELISA, flow cytometry, and western blot. Our data demonstrated the important role of PF in protecting from hyperglycemia fluctuation-induced vascular injuries by minimizing inflammation and oxidative stress release and repression of the PKC*β*1 level.

## 2. Materials and Methods

### 2.1. HUVEC Culture

HUVECs were obtained from the healthy human umbilical cords as described in Baudin et al. [24]. The cells were incubated for 8 days under one of the following seven glycemic conditions as previously described [8]: (1) constant normal glucose medium (5.56 mmol/L), (2) constant high-glucose medium (25 mmol/L), (3) constant high-glucose medium with 1 h pretreatment with LY333531 (PKC*β*1 inhibitor), (4) alternating normal and high-glucose medium (5.56/25 mmol/L) every 24 h, (5) alternating normal and high-glucose medium (5.56/25 mmol/L) every 24 h with 1 h pretreatment with LY333531, (6) alternating normal and high-glucose medium every 24 h with daily PF (100 *μ*M, Dalian Meilun Biotechnology, China) treatment, and (7) alternating normal and high-glucose medium every 24 h with daily metformin hydrochloride (MH) (1 mM) treatment.

### 2.2. Establishment of a Rat Model of Intermittently High Glucose

Sprague Dawley (SD) male rats weighing 170-190 g were obtained from the National Institutes for Food and Drug Control (SCXK 2009-0017) and housed in an animal facility at Xiyuan Hospital, China Academy of Chinese Medical Sciences, according to the guidelines for laboratory animals approved by the Beijing Experimental Animal Management Center. After 2 weeks of adaptation, the normal diet was switched to a high-fat and high-glucose diet containing 20% fat from lard with 1.25% (wt/wt) cholesterol and 10% (wt/wt) sucrose purchased from Huafukang Feed Company, Beijing, China. Four weeks later, rats were injected intraperitoneally with streptozotocin (STZ) at 30 mg/kg. An Ascensia BRIO blood glucose monitoring system (Bayer company, Germany) was used to measure plasma glucose levels, including the fasting state and 2 h postprandial blood glucose levels 3 days post-STZ injection. Rats with marked hyperglycemia (fasted blood glucose level greater than 11.1 mmol/L and postprandial blood glucose level > 33.3 mmol/*L*) after 3 days of administration of STZ were confirmed to have DM [25]. Fifty-two DM rats were divided into four groups according to blood glucose levels (the mean amplitude of glycemic excursion (MAGE) and the largest amplitude of glycemic excursion (LAGE)): (1) stable high blood glucose (SHG, *n* = 11) group, fed with low glycemic diet (Beijing Nuokangyuan Biotechnology) by gavage, (2) intermittent high blood glucose (IHG, *n* = 11) group, fed with high glycemic index diet (Beijing Nuokangyuan Biotechnology) by gavage, (3) PF-treated (PF, *n* = 10) group, fed with high glycemic index diet and received the treatment of PF (Dalian Meilun Biotechnology, China; 0.01 g/kg) by gavage, and (4) MH-treated (*n* = 10) group, fed with high glycemic index diet and received the treatment of MH (Dalian Meilun Biotechnology, China; 0.15 g/kg) by gavage. Ten untreated SD rats fed with normal diet, which only received PBS treatment by gavage, were used as a control group. Rats in these five groups were fed three times per day for 1 h and maintained for six weeks.

Establishment of the intermittently high glucose rat model was evaluated by measuring fasting blood glucose, triglyceride levels, fasting insulin levels, insulin resistance, and glucose variability using MAGE and LAGE, and the morphology of aortic roots with hematoxylin and eosin (H&E) staining. Fasting blood glucose (FBG) and postprandial 2 h blood glucose levels were determined for 5 days after feeding on low or high glycemic index forage for four weeks. Insulin resistance was evaluated using the homeostasis model assessment estimate of insulin resistance (HOMA-IR) [26, 27] with the following formula: fasting insulin level (*μU*/mL) × fasting blood glucose (mmol/*L*)/22.5. MAGE used for assessing the intraday glucose variability was calculated by measuring the arithmetic mean of the differences in consecutive peaks and nadirs, which were taken into consideration only if they exceeded the standard deviation from the mean. LAGE was defined as the maximal sensor glucose levels minus the minimal daily sensor glucose levels [28].

### 2.3. Cell Apoptosis Assay Using Flow Cytometry

To unveil the impact of fluctuant hyperglycemia on cell injury, HUVECs were subjected to different glycemic conditions followed by apoptosis analysis using flow cytometry. Annexin V-FITC/PI staining was performed to measure apoptosis using flow cytometry according to the manufacturer's protocol. Briefly, 1 × 10^6^ HUVECs were collected, washed with PBS, and resuspended in 100 *μ*L 1× binding buffer, followed by addition of 5 *μ*L Annexin V-FITC. After incubation for 15 min at 37°C, the buffer was removed by centrifugation, and the cells were resuspended in a reaction buffer containing 5 *μ*L propidium iodide (PI), followed by flow cytometry analysis. All reagents were purchased from BD Biosciences.

### 2.4. ELISA Assay and Western Blotting Analysis

To further verify the potential role of intermittent high glucose in the inflammatory response and oxidative stress, we determined the contents of tumor necrosis factor-*α* (TNF-*α*), platelet endothelial cell adhesion molecule (PECAM-1), advanced oxidation protein products (AOPPs), and total antioxidant capacity (T-AOC) in the supernatant from HUVEC culture and in sera of DM rats using ELISA kits (Westang Bio-Tech, Shanghai, China) according to the manufacturer's protocols. Serum von Willebrand factor (VWF) and soluble E-selectin (sE-selectin) levels were also determined as the endothelial injury indexes in rats by ELISA. The protein levels of PKC*β*1 in HUVECs and rat aorta were determined using western blotting analysis according to the standard procedure as previously described [29]. Glyceraldehyde-3-phosphate dehydrogenase (GAPDH) was used as a loading control.

### 2.5. Statistical Analyses

SPSS software version 16.0 (SPSS Inc., Chicago, IL, USA) was used for statistical analysis. Data were analyzed using one-way ANOVA with multiple comparison tests and presented as mean ± standard deviation (SD). The normality was checked using the Shapiro-Wilk test. The difference between groups was tested by least significant difference (LSD), and the Levene method was used for homogeneity test of variance. *P* < 0.05 was considered statistically significant.

## 3. Results

### 3.1. Effect of Hyperglycemia Fluctuation on the Apoptosis of HUVECs

**Figure 2:**
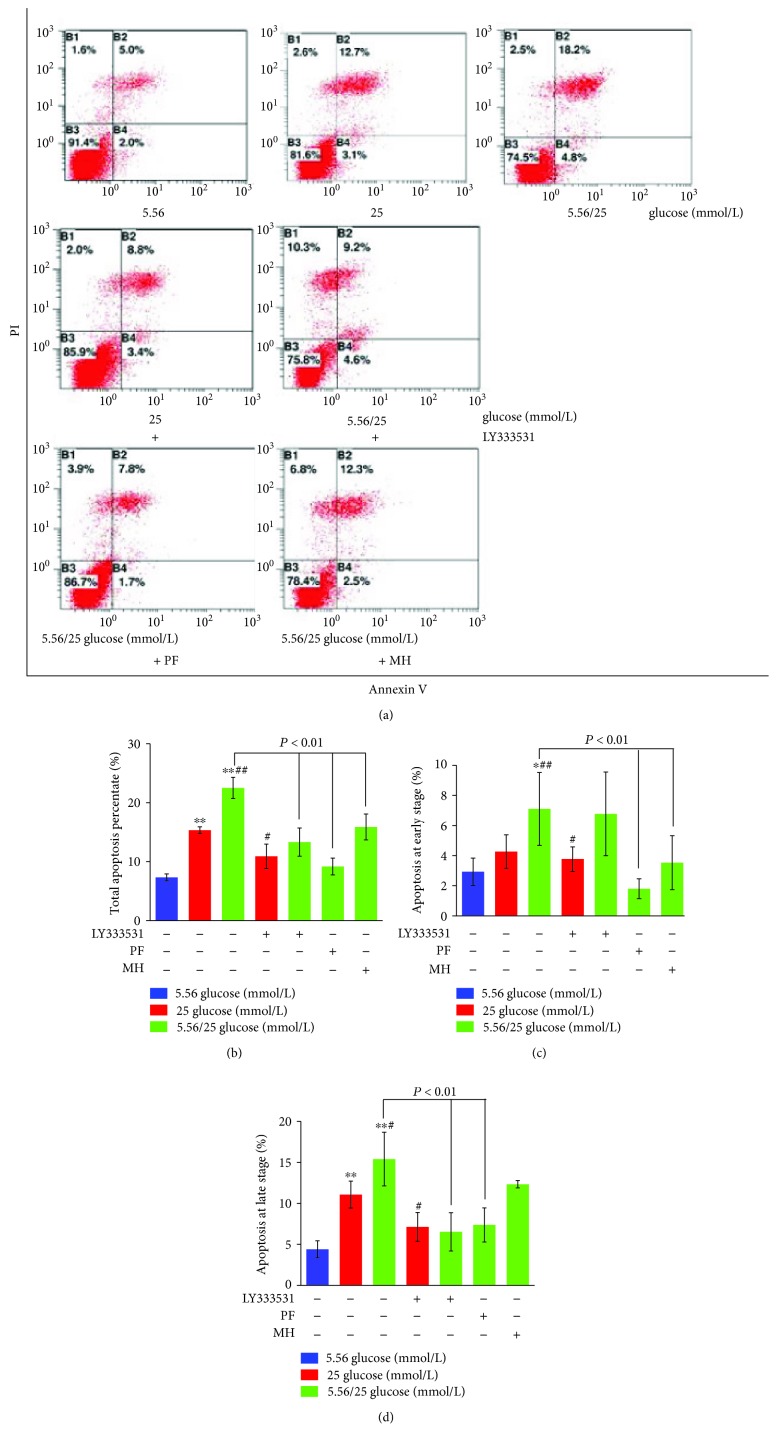
Effects of paeoniflorin (PF) on the apoptosis rate of human umbilical vein endothelial cells (HUVECs) cultured with different concentrations of glucose for 8 days. HUVECs were cultured in the presence of normal (5.56 mmol/L), high (25 mmol/L), or alternating normal/high concentrations as described in Materials and Methods and treated with LY333531, PF, or metformin hydrochloride (MH). Representative flow cytometry scatter plots of the total percentage of apoptosis are shown in (a). The total percentage of apoptosis was reflected by its fluorescent intensity (b). Early (c) and late (d) apoptosis was determined in the various groups. Data are presented as mean ± standard deviation (*n* = 6). ^∗^*P* < 0.05 and ^∗∗^*P* < 0.01 vs. glucose 5.56 mmol/L; ^#^*P* < 0.05 and ^##^*P* < 0.01 vs. 25 mmol/L.

As shown in Figure 2, after 8 days, a large number of apoptotic cells were observed when cultured with fluctuating glucose levels. Stable high glucose levels significantly increased the total apoptosis rate during the whole period (Figures 2(a) and 2(b); *P* < 0.01) and at late stage (Figure 2(d); *P* < 0.01), but only an increasing tendency at early stage (Figure 2(c)), when compared with normal glucose concentration (5.56 mmol/L glucose). Surprisingly, intermittent high glucose levels significantly enhanced this apoptotic process (Figures 2(b)–2(d); *P* < 0.05 or 0.01). LY333531 treatment decreased the apoptosis rates both at the late stage and at the whole stage, but not at the early stage, induced by the stable and intermittent high glucose (Figures 2(d) and 2(a); *P* < 0.05 or 0.01). As expected, PF treatment significantly inhibited intermittent high glucose-induced cell apoptosis at the early, late, and whole stages (*P* < 0.01 vs. 5.56/25 mmol/L glucose).

### 3.2. Inflammation, Oxidative Stress, and PKC*β*1 Protein Levels in Hyperglycemia Fluctuation of HUVECs

**Figure 3:**
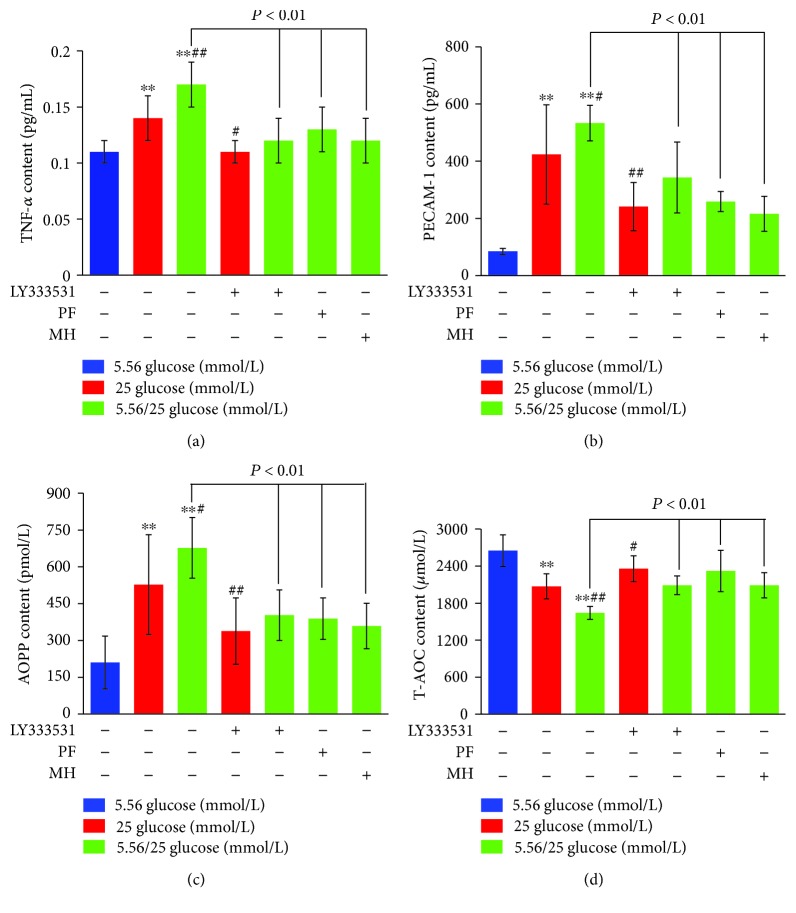
Effects of paeoniflorin (PF) on inflammatory factor and oxidative stress levels in human umbilical vein endothelial cells (HUVECs) under different concentrations of glucose for 8 days. HUVECs were cultured in the presence of normal (5.56 mmol/L), high (25 mmol/L), or alternating normal/high concentrations, as described in Materials and Methods. The levels of TNF-*α* (a), PECAM-1 (b), AOPPs (c), and T-AOC (d) in HUVECs induced by different pretreatments were determined in the various groups. Data are presented as mean ± standard deviation. ^∗^*P* < 0.05 and ^∗∗^*P* < 0.01 vs. glucose 5.56 mmol/L; ^#^*P* < 0.05 and ^##^*P* < 0.01 vs. 25 mmol/L. MH: metformin hydrochloride.

As shown in Figure 3(a), TNF-*α* levels increased under stable high glucose and intermittent high glucose compared to the normal glucose group (*P* < 0.01). The level of TNF-*α* under intermittent high glucose further significantly increased compared to the stable high glucose group (*P* < 0.01). In contrast, LY333531 pretreatment reduced TNF-*α* levels by 21.43% under stable high glucose and 29.41% under alternating 5.56/25 mmol/L glucose, respectively, as compared to nontreated cells under the corresponding conditions (Figure 3(a)). PF and MH pretreatment decreased the levels of TNF-*α* by 23.53% and 29.41%, respectively, in HUVECs cultured with alternating 5.56/25 mmol/L glucose (Figure 3(a)). Similarity, we observed the increased protein levels of PECAM-1 and AOPPs in stable high glucose (>5-fold and >2-fold, *P* < 0.01) and intermittent high glucose (>6-fold and >3-fold, *P* < 0.01), compared to the normal glucose group, while the levels of PECAM-1 and AOPPs in stable high glucose (75.62% and 56.11%, *P* < 0.01) and intermittent high glucose (55.44% and 68.10%, *P* < 0.01) with inhibition of PKC*β*1 were decreased compared to those of the corresponding conditions (Figures 3(b) and 3(c)). T-AOC level was decreased in stable high glucose by 27.94% (*P* < 0.01) and intermittent high glucose by 61.40% (*P* < 0.01) when compared to the normal glucose group, with further significant decrease in intermittent high glucose (Figure 3(d)). The effect of PKC*β*1 inhibition by LY333531 and PF on inflammation and oxidative stress was similar to the HUVECs treated with MH (*P* < 0.01 vs. 5.56/25 mmol/L glucose).

**Figure 4:**
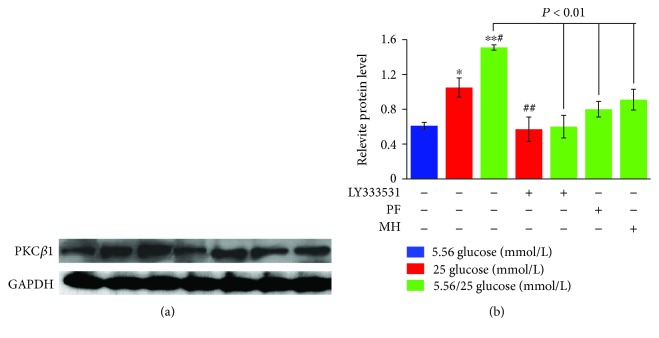
Western blot analysis of PKC*β*1 in the supernatant of human umbilical vein endothelial cells under different glucose concentrations. The band intensity was normalized to that of glyceraldehyde-3-phosphate dehydrogenase (GAPDH) (a), and the average band intensity after normalization is presented as a bar graph (b). ^∗^*P* < 0.05 and ^∗∗^*P* < 0.01 vs. glucose 5.56 mmol/L; ^#^*P* < 0.05 and ^##^*P* < 0.01 vs. 25 mmol/L. PF: paeoniflorin; MH: metformin hydrochloride.

As shown in Figure 4, PKC*β*1 protein levels in HUVECs under both stable and alternating high glucose conditions increased significantly as compared to the normal glucose condition (*P* < 0.05 and 0.01, respectively). In addition, the protein level of PKC*β*1 in alternating high glucose condition was significantly increased by 43.81% (*P* < 0.05) compared to those of the stable high glucose. In contrast, pretreatment with LY333531, PF, and MH blocked the increase in PKC*β*1 protein levels under the stable or alternating high glucose conditions.

### 3.3. Establishment of a Rat Model of Hyperglycemia Fluctuation

**Figure 5:**
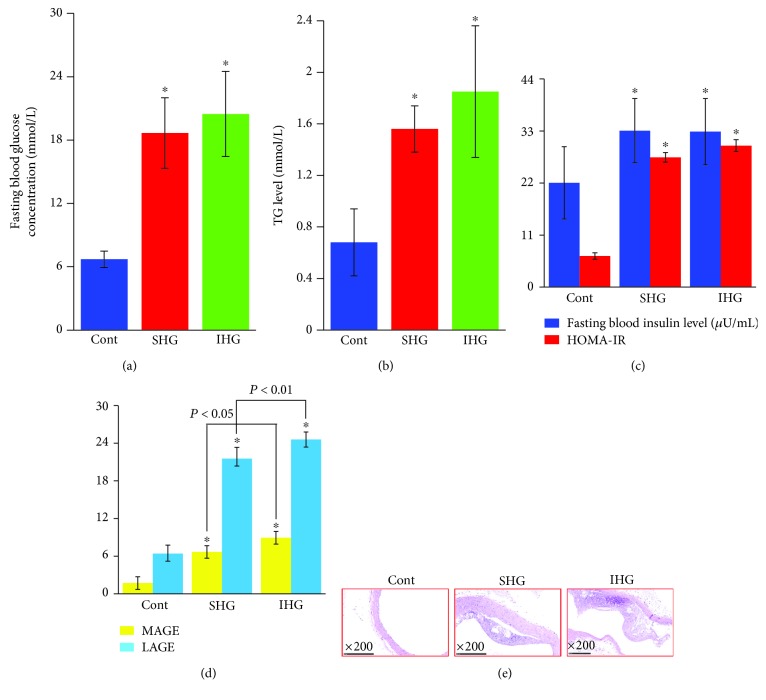
Establishment of a blood glucose fluctuation rat model. (a) Fasting blood glucose concentration, (b) triglyceride (TG) levels, (c) fasting blood insulin level and homeostasis model assessment estimate of insulin resistance (HOMA-IR) index, and (d) mean amplitude glycemic excursions (MAGE) and largest amplitude glycemic excursions (LAGE). ^∗^*P* < 0.01 compared with control rats. (e) Representative images of histological examination of aorta in rats by H&E staining with 200x magnification. Cont: control; SHG: stable high blood glucose; IHG: intermittent high blood glucose.

FBG (Figure 5(a)), TG (Figure 5(b)), fasting insulin, and HOMA-IR (Figure 5(c)) levels increased in rats fed on low or high glycemic index diet compared to rats fed on a normal diet (*P* < 0.01), indicating the successful establishment of the DM model of rats with insulin resistance. As shown in Figure 5(d), MAGE and LAGE markedly increased in rats fed on low or high glycemic index diet compared to rats fed a normal diet (*P* < 0.01). Furthermore, these two indices in rats fed on high glycemic index diet increased significantly compared to those in rats fed on low glycemic index diet (*P* < 0.05 and 0.01, respectively). As a result, a thickened aortic intimal layer and atherosclerosis plaque formation were observed in rats fed on low or high glycemic index diet (Figure 5(e)).

### 3.4. Endothelial Function, Inflammatory Cytokines, Oxidative Stress, and PKC*β*1 Protein Levels in DM Rats with Hyperglycemia Fluctuation

**Figure 6:**
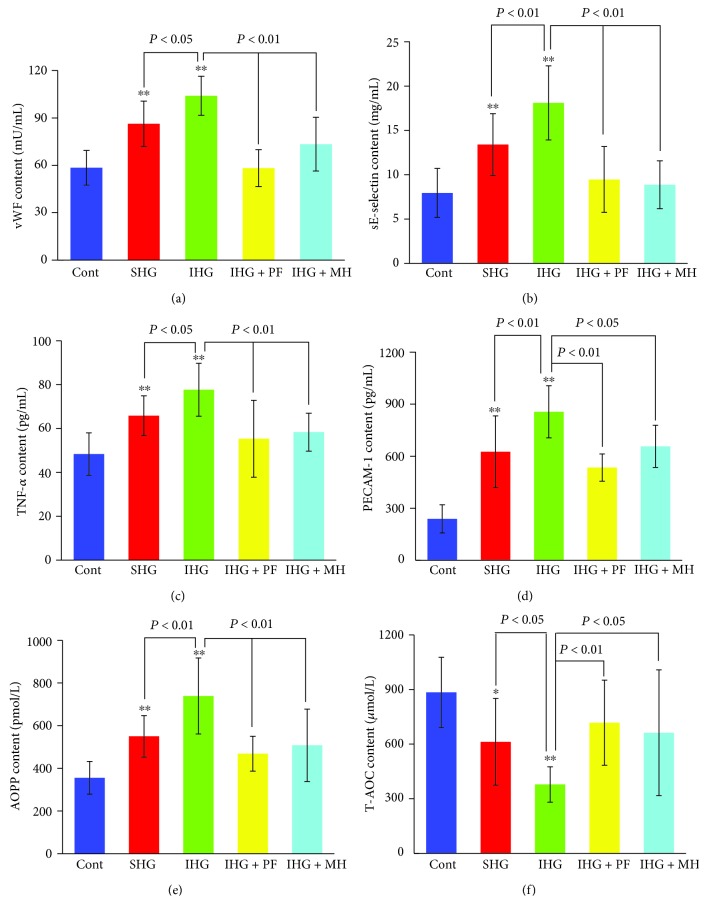
Effects of paeoniflorin (PF) on endothelial injury, inflammatory factors, and oxidative stress levels in rats fed with different diets for 4 weeks. Rats in the SHG and IHG groups were fed with low and high glycemic diet, respectively. von Willebrand factor (VWF) (a), sE-selectin (b), TNF-*α* (c), PECAM-1 (d), AOPPs (e), and T-AOC (f) levels were determined in the various groups. Data are presented as mean ± standard deviation. ^∗^*P* < 0.05 and ^∗∗^*P* < 0.01 compared with the control rats. Cont: control; SHG: stable high blood glucose; IHG: intermittent high blood glucose; MH: metformin hydrochloride.

**Figure 7:**
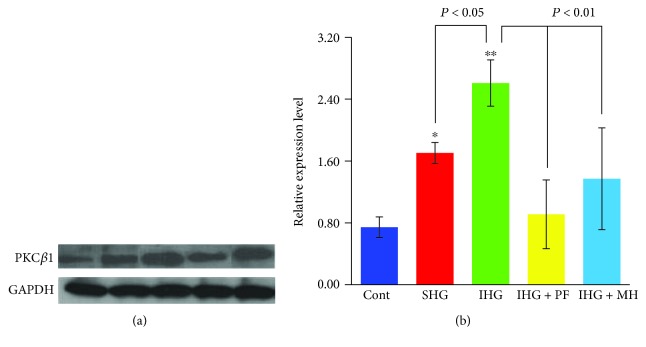
Western blot analysis of PKC*β*1 levels in rats fed with different diets for 4 weeks. Rats in the SHG and IHG groups were fed with a low and high glycemic diet, respectively. Signal intensity was normalized to that of glyceraldehyde-3-phosphate dehydrogenase (GAPDH) (a), and the average signal intensities after normalization are shown as a bar graph (b). ^∗^*P* < 0.05 and ^∗∗^*P* < 0.01 compared with the control rats. Cont: control; SHG: stable high blood glucose; IHG: intermittent high blood glucose; PF: paeoniflorin; MH: metformin hydrochloride.

As shown in Figure 6, the serum levels of VWF, sE-selectin, TNF-*α*, PECAM-1, and AOPPs significantly increased, whereas T-AOC levels significantly decreased in SHG and particularly in IHG groups compared to the control (*P* < 0.05 and *P* < 0.01, respectively). Pretreatment with both PF and MH attenuated such changes (Figure 6), consistent with the *in vitro* observations (Figure 3). Similarly, as shown in Figure 7, PKC*β*1 protein levels in the aorta increased in rats with low or high glycemic diet compared to control rats (*P* < 0.05 and *P* < 0.01, respectively), and pretreatment with PF and MH blocked the increase in PKC*β*1 protein levels induced in SHG and IHG groups.

## 4. Discussion

Vascular disorders, especially cardiovascular disorders, are major causes of morbidity and mortality in diabetic patients [30]. Vascular endothelial cell injury and dysfunction greatly contribute to the occurrence and development of DM complications [31]. Compared to persistent hyperglycemia, fluctuant hyperglycemia has more capacity to increase microvascular lesions and the risk of cardiovascular death [32], even though the underlying molecular mechanisms are still elusive. To figure out the mechanisms of endothelial dysfunction induced by fluctuant hyperglycemia, HUVECs with exposure to intermittent glucose and DM rat models with fluctuating hyperglycemia were used in our study.

A deleterious effect of glucose fluctuations for HUVECs has been reported. Periodic high glucose concentration increases apoptosis of HUVECs in terms of cell cycle analysis, DNA fragmentation evaluation, and the levels of Bcl-2 and Bax measurements in comparison with stable high glucose concentration [5]. Consistently, our data also found that hyperglycemia fluctuation led to exacerbation of the apoptotic effect on HUVECs. Oxidative stress generation [33], inflammation [34], and PKC activation [35] have been proposed as the factors for the damaging effect of hyperglycemia. Piconi et al. suggested that both stable and oscillating hyperglycemia increased HUVEC apoptosis through ROS overproduction at the mitochondrial electron transport chain [36]. In our study, levels of TNF-*α* and PECAM-1 were measured as inflammation cytokines, while AOPPs and T-AOC were used as oxidative stress indicators. Our findings demonstrated that high glucose induced an increase in TNF-*α*, PECAM-1, and AOPPs in HUVECs, which was accompanied by a decrease in T-AOC. These effects were amplified during glucose fluctuations. Previous study has demonstrated that PKC activation and its induced HAD(P)H oxidase participate in apoptosis of HUVECs exposed to glucose fluctuations [37]. To verify the role of PKC*β*1 involved in endothelial dysfunction caused by glucose fluctuations, a PKC*β* isoform selective inhibitor (LY333531) was used in our study. LY333531, or ruboxistaurin, is reported to treat diabetic retinopathy and nephropathy [38]. Upregulation of PKC*β*1 protein was shown in HUVECs cultured with both stable and intermittent high glucose concentration, which was more pronounced in intermittent high glucose. However, a remarkably decreased expression of PKC*β*1 protein induced by LY333531 pretreatment was associated with a significantly decreased apoptosis and an inhibited expression of TNF-*α*, PECAM-1, and AOPPs, but with a simultaneous increase in T-AOC. Thus, cumulating *in vitro* evidence suggested that glucose fluctuation was more deleterious to HUVECs than constant high glucose by intensifying apoptosis, aggravating inflammation activation, increasing oxidative stress indicators, and elevating PKC*β*1 expression.

Previous studies have focused on *in vitro* experiments and rarely on *in vivo*. In our study, the DM rat model with fluctuating hyperglycemia treatment was used to further verify the findings in HUVECs. VWF and sE-selectin were used as biomarkers of endothelial injury. High glucose caused increased levels of VWF, sE-selectin, TNF-*α*, PECAM-1, and AOPPs in sera and high PKC*β*1 protein levels in the aorta, which was accompanied by a declined serum level of T-AOC.

Paeoniflorin, a monoterpene glucoside, is the main active ingredient of the root of *P. lactiflora* and has multiple biological activities such as antioxidation, cognition-enhancing, anticoagulation, anticonvulsant, vasodilation, mild sedation, and potent analgesia [39, 40]. Till now, the protective roles of PF refer to amelioration of DM or its complications, such as diabetic nephropathy [21, 41], and cardiovascular events [19], nothing about PF and hyperglycemia fluctuation. In this study, it demonstrated that PF could alleviate vascular injuries induced by hyperglycemia fluctuations in terms of the release of TNF-*α*, PECAM-1, oxidative stress, and the expression of PKC*β*1 in HUVECs subjected to intermittent glucose levels and in DM rats with fluctuating hyperglycemia. Metformin is verified to have beneficial effects on diabetes patients, especially for diabetes-initiated vascular disease, by a reduction in both oxidative stress and chronic inflammation [23]. Since PF showed a similar role as metformin, we demonstrated that PF exhibited a protective role during hyperglycemia fluctuation. Whether PF in combination with metformin may have better clinical efficacy, or PF can be better to alleviate the side effect of metformin, awaits further investigation.

In summary, our study suggests that fluctuant high glucose induces vascular endothelial injury by elevating inflammatory cytokines and aggravating oxidative stress. In addition, PKC*β*1 might play a vital role in these intermittent hyperglycemia state-induced damages. Our data show that paeoniflorin ameliorates vascular endothelial injuries through antioxidative and anti-inflammatory effects and decreasing the PKC*β*1 protein level.

## Data Availability

The data used to support the findings of this study are included within the article.

## Abbreviations

PF: Paeoniflorin
DM: Diabetes mellitus
HUVECs: Human umbilical vein endothelial cells
PKC*β*1: Protein kinase C beta 1
SHG: Stable high glucose
IHG: Intermittent high glucose
MH: Metformin hydrochloride
MAGE: Mean amplitude glycemic excursion
LAGE: Largest amplitude glycemic excursion
GI: Glucose index.
